# PULMOEAST: A Comprehensive Analysis of Pulmonary Hypertension in Eastern India

**DOI:** 10.7759/cureus.50996

**Published:** 2023-12-23

**Authors:** Anil K Singhi, Soumya K Mohapatra, Nandini Biswas, Kasturi H Bandyopadhyay, Sanjay Bhalerao, Anish Nath

**Affiliations:** 1 Pediatric and Congenital Heart Disease, Medica Super Specialty Hospital, Kolkata, IND; 2 Pulmonary Medicine, Medica Super Specialty Hospital, Kolkata, IND; 3 Anesthesiology and Pain Medicine, Medica Super Specialty Hospital, Kolkata, IND; 4 Pediatric Intensive Care Unit, Vishesh Jupiter Hospital, Indore, IND

**Keywords:** collaborative care, comprehensive evaluation, challenges, patient outcomes, treatment modalities, clinical profiles, eastern india, pulmonary hypertension

## Abstract

Background

Pulmonary hypertension (PH) is a debilitating cardiovascular disorder characterized by abnormally elevated blood pressure within the lungs. The diverse range of causes and varied clinical presentations contribute to the complexity of its diagnosis and management. In eastern India and surrounding areas, awareness of PH remains limited, and resources for its management are scarce. This study aims to address this knowledge gap by investigating clinical characteristics and treatment approaches adopted for PH patients in eastern India.

Methods

This retrospective-prospective cohort study included patients diagnosed with PH, defined by a pulmonary artery systolic pressure (PASP) > 50 mmHg or a mean pulmonary artery pressure (mPAP) >20 mmHg, between July 2015 and October 2023. Data retrieved from hospital records formed the retrospective cohort, while the prospective cohort comprised patients directly recruited for the study.

Results

The PULMOEAST study enrolled 93 patients with confirmed PH, divided into prospective (59 patients) and retrospective (34 patients) cohorts. The most prevalent cause of PH was congenital heart disease (CHD), with shunt lesions (59.13%), followed by complex CHD (13.97%) and idiopathic PH (20.43%). Six additional patients presented with rare causes of PH, and three experienced transient PH following atrial septal defect device closure. Geographic distribution revealed that 72.04% of patients originated from eastern India, while 18.27% hail from other eastern states and 8.6% from neighboring countries. Patients exhibited varying functional classes (FC), with 57 classified as FC-II and 31 classified as FC-III. Treatment strategies primarily involve supportive medications and pulmonary vasodilators. Monotherapy was administered to 26 patients (27.95%), dual therapy to 50 patients (53.76%), and triple therapy to one patient. Notably, 16 patients did not receive any vasodilator therapy as they were waiting for further evaluation. Among the vasodilator regimen, two patients received Selexipag. Three patients underwent intervention for shunt lesion closure, including one patient who received a fenestrated atrial septal occluder implant. Additionally, one patient underwent clot removal for pulmonary thromboembolism. Despite the overall positive response to treatment, the study recorded eight fatalities (8.6%) during the observation period. However, most patients exhibited significant improvement, including a decrease in functional class, during a mean follow-up duration of 14.31 months.

Conclusion

The PULMOEAST study undertook a comprehensive exploration of PH in eastern India and surrounding regions, revealing a stark dominance of CHD as the primary culprit. The study confirmed the pivotal role of echocardiography as a readily available and effective tool for both initial and follow-up evaluations in resource-scarce settings. It painted a hopeful picture by showcasing significant clinical improvement in most treated patients, with supportive medications and pulmonary vasodilators playing a crucial role. However, the diverse etiologies, limited access to PH-specific resources, and lack of widespread awareness within the region continue to pose substantial challenges for patients. The study underscores the need for refined diagnostic approaches, cost-effective management strategies, collaborative care initiatives, and enhanced patient education to optimize PH care and improve outcomes in eastern India.

## Introduction

Pulmonary hypertension (PH) is a rare but significant global health concern characterized by an abnormally elevated mean pulmonary arterial pressure exceeding 20 mmHg. Although its estimated prevalence ranges from approximately 15 to 60 individuals per million, its rarity does not diminish its far-reaching impact. Delayed diagnosis of PH is common due to its non-specific symptoms, negatively affecting patient prognosis. Despite its uncommon occurrence, the relentlessly debilitating nature of PH poses a substantial risk of morbidity and potential mortality [1].

Recent classification systems categorize PH into five distinct groups, each associated with specific etiologies [2]. Accurate identification of the underlying cause remains crucial for optimal patient care. Various medications have demonstrated effectiveness in managing PH across diverse severity levels, addressing both primary and, to some extent, secondary cases [3].

Despite advancements in overall PH care, a notable lack of data exists concerning PH in the eastern region of India, similar to other low- and middle-income countries [1]. This study aims to analyze patients with pulmonary hypertension at a tertiary care facility in eastern India. Its objective is to deepen our understanding of the PH profile in this region, shedding light on current care approaches and prognostic outcomes.

## Materials and methods

Objectives

The core focus of this research was a meticulous examination of the clinical characteristics of patients grappling with pulmonary hypertension. Additionally, it assessed the prevalent treatment approaches and evaluated clinical outcomes at a tertiary healthcare facility in eastern India. Ultimately, this pursuit aimed to improve the quality of care administered to individuals afflicted by PH in the foreseeable future.

Study design and patient population

This observational study was conducted at the Department of Pediatric and Congenital Heart Disease at Medica Super Speciality Hospital, a tertiary-care center in eastern India. It incorporated both prospective and retrospective components. The analysis involved patients diagnosed with PH, according to specific criteria outlined below, from July 2015 to October 2023. The study protocol received approval from the institutional ethics and clinical research committee in November 2022 (details provided below).

The prospective cohort included patients assessed from November 2022 onward, encompassing first-time visitors and follow-up patients. The retrospective cohort comprised patients whose information was retrieved from the institution's electronic database between July 2015 and October 2022, excluding those actively receiving follow-up care after October 2022.

Definition of pulmonary hypertension

In this study, pulmonary hypertension (PH) was diagnosed by elevated pulmonary artery pressure, specifically exceeding 50 mmHg for pulmonary artery systolic pressure (PASP) or 20 mmHg for mean pulmonary artery pressure (mPAP), as determined by echocardiography or right heart catheterization, respectively [2-4].

The classification of PH grades was based on PASP: mild PH (35-50 mmHg), moderate PH (50-70 mmHg), and severe PH (>70 mmHg) [5]. Similarly, mPAP thresholds defined PH severity as mild PH (<30 mmHg), moderate PH (30-45 mmHg), and severe PH (>45 mmHg) [6].

Inclusion and exclusion criteria

This study included patients diagnosed with pulmonary hypertension (PH) based on an echocardiographic pulmonary artery systolic pressure (PASP) exceeding 50 mmHg or a mean pulmonary artery pressure (mPAP) exceeding 20 mmHg measured during cardiac catheterization. Patients with hyperkinetic PH in congenital heart disease (CHD) with normal pulmonary vascular resistance and persistent pulmonary hypertension of the newborn were excluded.

Ethical considerations

The study received Institutional Clinical Research and Ethics Committee (CREC) approval (CREC/2022/NOV/1-iv). Prospective elements adhered to the study protocol approved by the CREC and obtained informed consent from participants. Retrospective data were collected from hospital records, with CREC waiving patient consent for this cohort. Patient identities were anonymized to ensure privacy.

Patient data collection

Data collection procedures encompassed patient interviews (prospective cohort only), medical record reviews, and retrieval of archived data for all enrolled individuals. Comprehensive baseline demographic, clinical, imaging, and laboratory information, along with prescribed pulmonary hypertension medications, was gathered. Follow-up data were collected through clinical evaluations, hospital records, and phone calls to patients and their families.

Statistical analysis

Quantitative data were presented as mean values with standard deviations, while qualitative data were represented as frequencies and percentages. Data analysis was performed using Microsoft Excel software.

## Results

The study enrolled 93 patients diagnosed with PH, divided into two groups: 59 (63.44%) in the prospective cohort and 34 (36.55%) in the retrospective cohort. The mean patient age was 31.19 years, with a female predominance (Table 1). The majority of patients were from West Bengal (72.04%), followed by other eastern states (18.27%) and neighboring countries (8.6%) (Table 2). Given the patient distribution, the study aptly bears the name "PULMOEAST," signifying its focus on pulmonary hypertension in the eastern Indian subcontinent. 

**Table 1:** Demographic and clinical details of the patients n: total number of patients, n1: number of patients in prospective cohort, n2: number of patients in retrospective cohort, %: % of the patients in respective group. PASP: Pulmonary artery systolic pressure, mPAP: Mean pulmonary artery pressure, Qp:Qs: Pulmonary blood flow to systemic blood flow ratio, PVRI: Pulmonary vascular resistance index, CTPA: Computed tomographic pulmonary artery angiogram, SD: Standard deviation, SpO2: Saturation of peripheral oxygen

Parameter	Total cohort, n (%)	Group 1 (Prospective), n1 (%)	Group 2 (Retrospective), n2 (%)
Number of patients	93	59	34
Age in years, Mean ± SD	31.19 ± 19.68	30.30 ± 19.71	29.26 ± 19.78
Sex (Female)	48 (51.61%)	30 (50.84 %)	18 (52.94 %)
Weight in kilogram, Mean ± SD)	45.48± 18.83	45.16 ± 20.01	45.5 ± 17.02
Functional class-I	2 (2.15%)	1 (1.69 %)	1 (2.94 %)
Functional class-II	57 (61.29%)	40 (67.79 %)	17 (50 %)
Functional class-III	31 (31.33 %)	16 (27.11 %)	15 (44.11 %)
Functional class-IV	4 (4.30 %)	0 (0 %)	0 (0 %)
SpO_2_ (%), Mean ± SD	91.98 ± 7.24	92.29 ± 7.36	92.83. ± 6.12
PASP by echocardiogram in mm hg, Mean ± SD,	84.24 ±16.26	84.12. ± 15. 23	84.45. ± 18.18
Cardiac catheterization study	20 (21.50 %)	15 (25.42 %)	5 (14.70 %)
PASP during catheterization, mm hg, Mean ± SD	71.95 ± 27.03	80 ± 23.96	47.8 ± 2.12
mPAP during catheterization, mm hg, Mean ± SD	43.9 ± 18.06	49.69. ± 16.73	28.8 ± 12.37
Qp:Qs , Mean ± SD	1.55 ± 0.74	1.55. ± 0.82	1.55. ± 0.54
PVRI in wU.m2, Mean ± SD)	9.23 ± 6.56	10.66 ± 7.07	5.23. ± 2.02
CTPA	30 (32.25%)	19 (32.30 %)	11 (32.35 %)
Respiratory treatment	26 (27.95 %)	20 (33.89 %)	6 (17.64 %)
Follow up period in months, Mean ± SD	14.31 ±20.94 (6)	19.68. ± 24.13	5 ± 12.64

**Table 2:** Number of the patients residing in the different eastern Indian states and the neighboring countries n: total number of patients, n1: number of patients in the prospective cohort, n2: number of patients in the retrospective cohort, %: % of the patients in the respective group.

Residence	Total cohort, n (%)	Group 1 (Prospective), n1 (%)	Group 2 (Retrospective), n2 (%)
West Bengal	67 (72.04 %)	41 (69.49 %)	26 (76.47 %)
Jharkhand	7 (7.52 %)	5 (8.47 %)	2 (5.88 %)
Bihar	6 (6.45 %)	6 (10.16 %)	0 (0 %)
Northeast states	4(4.3 %)	2 (3.38 %)	2 (5.88 %)
Uttar Pradesh	1(1.07 %)	0 (0 %)	1 (2.94 %)
Neighboring country, Bangladesh	7(7.52 %)	5 (8.47 %)	2 (5.88 %)
Neighboring country, Bhutan	1(1.07 %)	0 (0 %)	1 (2.94 %)

The patients exhibited diverse PH etiologies: 59.13% had congenital heart disease (CHD) with shunt lesions, 13.97% had complex CHD, 20.43% had idiopathic PH, and 6.45% had rare causes, including systemic lupus erythematosus, pulmonary thromboembolism, pulmonary veno-occlusive disease, and lung diseases (Table 3). Functional class (FC) evaluation at presentation showed 57 patients in FC-II and 31 patients in FC-III. Echocardiographic assessment revealed an average PASP of 84.24 mm Hg and a mean oxygen saturation of ~92%. 

**Table 3:** Cause of pulmonary hypertension in the different groups of patients n: total number of patients, n1: number of patients in the prospective cohort, n2: number of patients in the retrospective cohort, %: % of the patients in the respective group. The pregnant patients were part of the congenital heart disease cohort.

Cause of pulmonary hypertension	Total cohort, n (%)	Group 1 (Prospective), n1 (%)	Group 2 (Retrospective), n2 (%)
Ventricular septal defect	16 (17.20 %)	8 (13.55 %)	8 (23.52 %)
Secundum atrial septal defect	28 (30.10 %)	19 (32.20 %)	9 (26.47 %)
Sinus venosus atrial septal defect	4 (4.30 %))	4 (6.77 %)	0 (0 %)
Patent ductus arteriosus	7 (7.52 %)	5 (8.47 %)	2 (5.88 %)
Complex congenital heart disease	13 (13.97 %)	8 (13.55 %)	5 (14.70 %)
Pregnancy with pulmonary hypertension (part of the congenital heart disease cohort)	3	1	2
Idiopathic pulmonary arterial hypertension	19 (20.43 %)	14 (23.72 %)	5 (14.70 %)
Systemic lupus erythematosus	2 (2.15 %)	1 (1.69 %)	1 (2.94 %)
Thromboembolic pulmonary hypertension	1 (1.07 %)	0 (0 %)	1 (2.94 %)
Lung disease	2 (2.15 %))	0 (0 %)	2 (5.88 %)
Pulmonary veno-occlusive disease	1 (1.07 %)	0 (0 %)	1 (2.94 %)

Twenty patients (21.5%) underwent cardiac catheterization, primarily within the institution, revealing an average PASP of 71.95 mm Hg and a mPAP of 43.9 mmHg. Additionally, the pulmonary to systemic blood flow ratio (Qp:Qs) was 1.55, and the calculated indexed pulmonary vascular resistance index (PVRI) stood at 9.23 wU.m². Furthermore, 32.25% underwent computed tomographic pulmonary artery angiogram (CTPA) for diagnostic imaging purposes, primarily to delineate cardiovascular anatomy, detect pulmonary thromboembolism, and assess lung diseases.

Limited data were available for N-terminal pro-B-type natriuretic peptide (NT pro BNP) evaluations (12 patients) and six-minute walk tests (six patients) within the prospective cohort due to recent implementation. Treatment involved pulmonary vasodilators, either as monotherapy (27.95%), dual therapy (53.76%), or no vasodilators, as prescribed in 17.2% of patients pending evaluation. A comprehensive overview of medications prescribed for various patient groups is presented in Table 4. Respiratory consultations done for 27.95% of patients, combined with cardiac care, notably improved clinical response and medication compliance. Three patients underwent shunt lesion closure during the study period, with one patient undergoing fenestrated atrial septal occluder placement. 

**Table 4:** Medications for different group of pulmonary hypertension patients n: total number of patients, n1: number of patients in the prospective cohort, n2: number of patients in the retrospective cohort, %: % of the patients in the respective group.

Medication	Total cohort, n (%)	Group 1 (Prospective), n1 (%)	Group 2 (Retrospective), n2 (%)
Sildenafil	18 (19.35 %)	9 (15.25 %)	9 (26.47 %)
Sildenafil + Bosentan	10 (10.75 %)	4 (6.77 %)	6 (17.64 %)
Sildenafil + Ambrisentan	12 (12.90 %)	8 (13.55 %)	4 (11.76 %)
Tadalafil	8 (8.60 %)	6 (10.16 %)	2 (5.88 %)
Tadalafil + Ambrisentan	27 (29.03 %)	23 (38.98 %)	4 (11.76 %)
Tadalafil + Ambrisentan + Selexipag	1 (1.07 %)	1 (1.69 %)	0 (0%)
Tadalafil + Selexipag	1 (1.07 %)	1 (1.69 %)	0 (0%)
No pulmonary vasodilator on initial evaluation (Pending detail evaluation)	16 (17.20 %)	7 (11.86 %)	9 (26.47 %)
Diuretics	49 (52.68 %)	36 (61.01 %)	13 (38.23 %)
Torsemide and Spironolactone	34 (36.55 %)	28 (47.45 %)	6 (17.64 %)
Furosemide and Spironolactone	15 (16.12 %)	8 (13.55 %)	7 (20.58 %)
Beta Blocker	28 (30.10 %)	20 (33.89 %)	8 (23.52 %)
Warfarin	11 (11.82 %)	11 (18.64 %)	0 (0%)
Aspirin	21 (22.58 %)	17 (28.81 %)	6 (17.64 %)
Aspirin + Warfarin	3 (3.22 %)	1 (1.69 %)	2 (5.88 %)
Digoxin	15 (16.12 %)	11 (18.64 %)	4 (11.76 %)
Nifedipine	3 (3.22 %)	2 (3.38 %)	1 (2.94 %)

To provide insights into the demographics and clinical presentations of PH patients in eastern India, six illustrative patient stories are highlighted:

Case 1: thromboembolic pulmonary hypertension: prompt recognition and resolution

**Figure 1:**
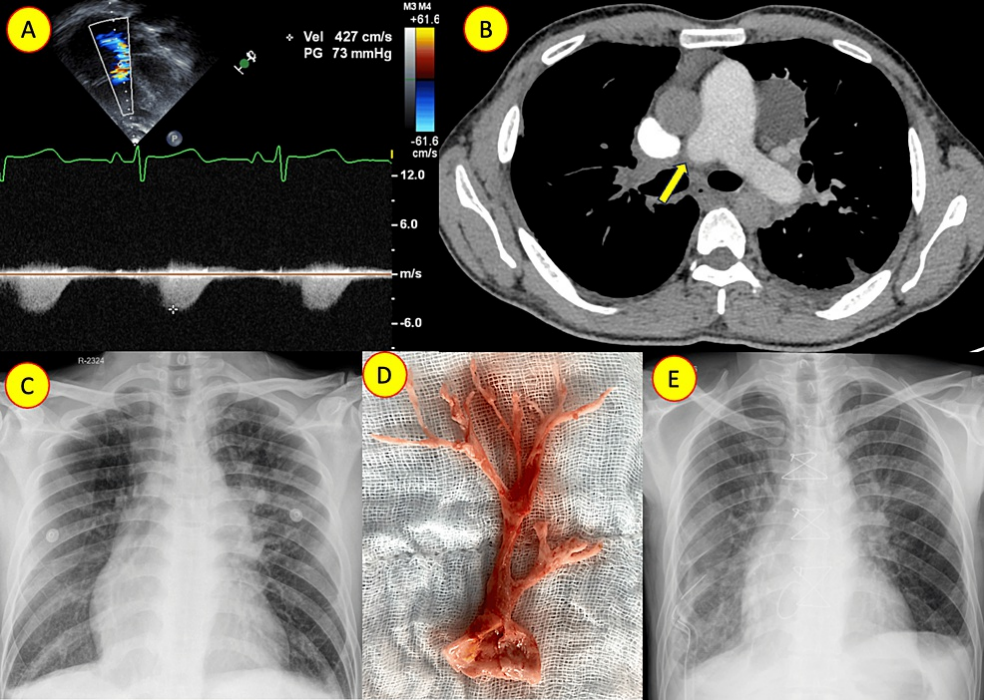
Pulmonary artery thromboembolism A: Continuous-wave Doppler revealing severe elevation of right ventricular systolic pressure, determined by the tricuspid regurgitation jet. B: Computed tomography pulmonary angiography demonstrating complete occlusion of the right pulmonary artery (yellow arrow). C: Chest X-ray (anterior-posterior view) exhibiting signs of differential vascularity and reduced right lung blood flow. D: A large thrombus recovered after removal. E: Chest X-ray (anterior-posterior view) post-thrombus removal, showing restored right lung blood flow.

A 36-year-old man from Bhutan presented with acute respiratory distress and was diagnosed with severe PH. A detailed evaluation revealed a substantial thrombus in the right pulmonary artery, necessitating surgical embolectomy (Figure 1). Following the procedure, the patient experienced complete recovery, with pulmonary artery pressure reduced to mild levels. Thromboembolic PH, a recognized cause, requires prompt evaluation and management. 

Case 2: pulmonary hypertension due to pulmonary veno-occlusive disease

**Figure 2:**
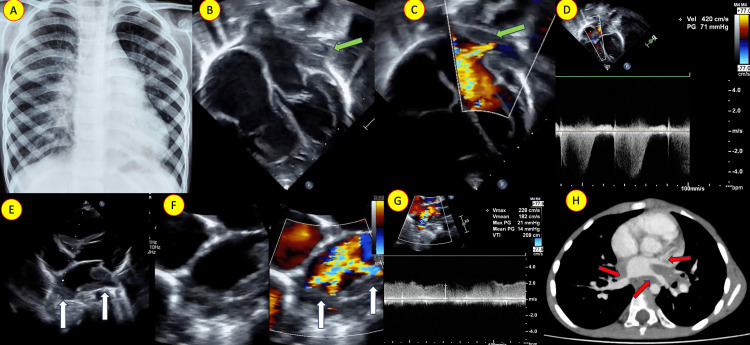
Pulmonary vein obstruction A: Chest X-ray (posterior-anterior view) indicating pulmonary venous congestion. B, C: Echocardiogram in four-chamber view showcasing dilated right heart chambers and obstruction in the left pulmonary vein by external tissue (green arrow). D: Continuous wave Doppler of the tricuspid regurgitation jet demonstrates elevated right ventricular systolic pressure. E, F: Parasternal short-axis view with color Doppler displaying obstructed pulmonary veins by soft tissue (white arrow). G: Doppler shows a significantly elevated mean gradient across the pulmonary vein. H: Computed tomography angiography depictis diffuse soft tissue (red arrow) in the mediastinum encasing the pulmonary veins, causing obstructions.

A seven-year-old boy from Bangladesh, previously diagnosed with severe PH, exhibited symptoms of effort intolerance and fatigue. A detailed assessment at our center revealed severe narrowing of the pulmonary veins due to mediastinal fibrosis (Figure 2). Consequently, he was referred to an oncology team for further care. These cases underscore the diverse etiologies and clinical presentations of PH in eastern India and emphasize the importance of comprehensive diagnostic evaluation and prompt management. 

Case 3: atrial septal defect closure with custom-made fenestrated occluder

**Figure 3:**
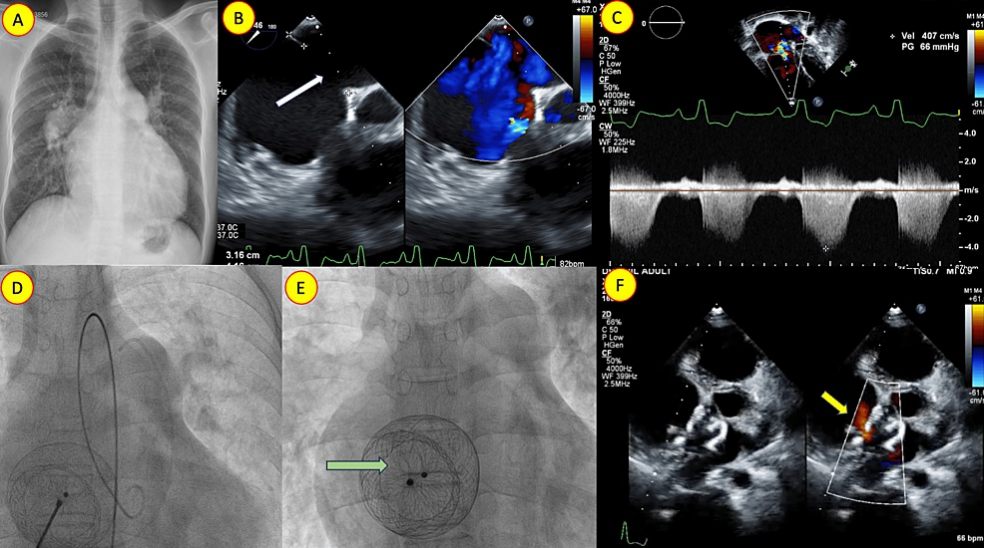
Secundum atrial septal defect closure with a fenestrated septal occluder A: Chest X-ray (posterior-anterior view) illustrating cardiomegaly and a dilated main pulmonary artery. Evidence of increased pulmonary blood flow is observed. B: Transesophageal echocardiogram with color Doppler revealing a large secundum atrial septal defect (ASD) predominantly shunting left to right (white arrow). C: Continuous-wave Doppler of the tricuspid regurgitation jet demonstrating elevated right ventricular systolic pressure. D, E: Hemodynamic assessment before and after ASD device deployment with a custom-made fenestration in the fluoroscopic image (green arrow). F: Echocardiogram in parasternal short-axis view with color Doppler showing the ASD device. The fenestration is seen shunting left to right (yellow arrow).

A 38-year-old man was diagnosed with an atrial septal defect (ASD) and severe pulmonary hypertension (PH), characterized by elevated pulmonary vascular resistance (PVR) and increased pulmonary blood flow. He underwent ASD device closure with a custom-made fenestration, resulting in symptom improvement. This case exemplifies an adapted approach to managing ASD and severe PH in the presence of both elevated PVR and increased pulmonary blood flow (Figure 3). 

Case 4: atrial flow regulator implantation for Idiopathic pulmonary hypertension

**Figure 4:**
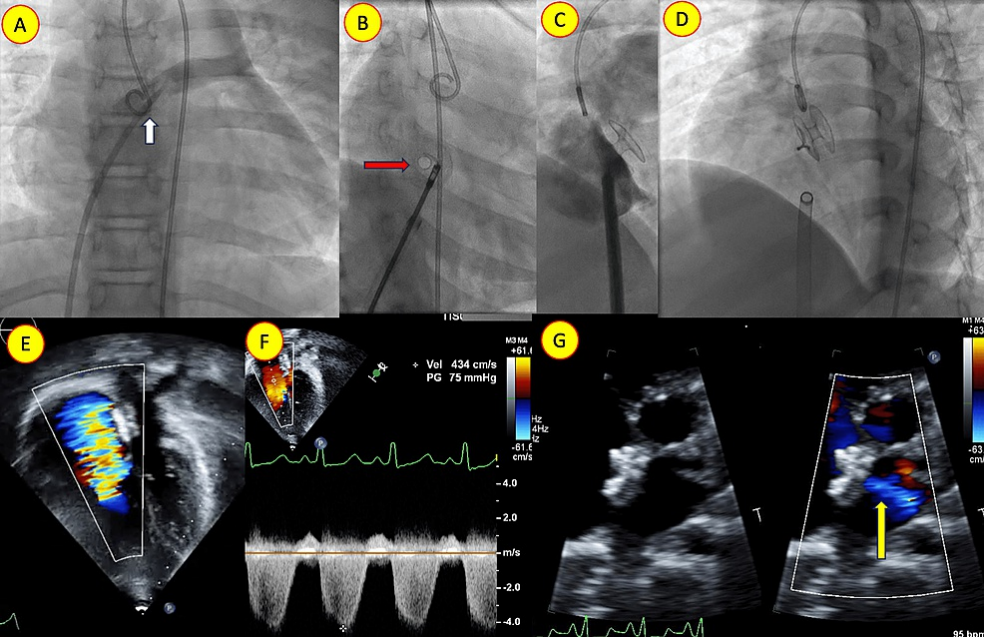
Atrial flow regulator implantation A-D: Fluoroscopic images illustrating the implantation sequence of atrial flow regulation implantation. A: Anteroposterior view showing septal puncture of the interatrial septum (white arrow). B: Oblique view after AFR deployment demonstrating fenestration (red arrow). C: Oblique view showing AFR deployment. D: post-release image showing stable device position. E-G: Follow-up echocardiogram displaying significant tricuspid regurgitation and elevated right ventricular systolic pressure by TR jet. The fenestration is flowing right to left, as seen in the modified parasternal view (G, yellow arrow).

A 15-year-old boy from the city was referred for syncope evaluation. He had severe pulmonary hypertension despite a structurally normal heart, except for right heart dilatation. An extensive investigation yielded no specific cause for severe PH. Eventually, the patient received an atrial flow regulator implantation in another organization, resulting in a right-to-left shunt at the atrial level and symptomatic relief. He is doing well with dual pulmonary vasodilators (Figure 4). This case history highlights the beneficial role of the atrial flow regulator in symptomatic idiopathic PH patients with an intact atrial septum.

Case 5: atrial septal defect, diastolic dysfunction, and severe pulmonary hypertension: Hemodynamic evaluation guides management

**Figure 5:**
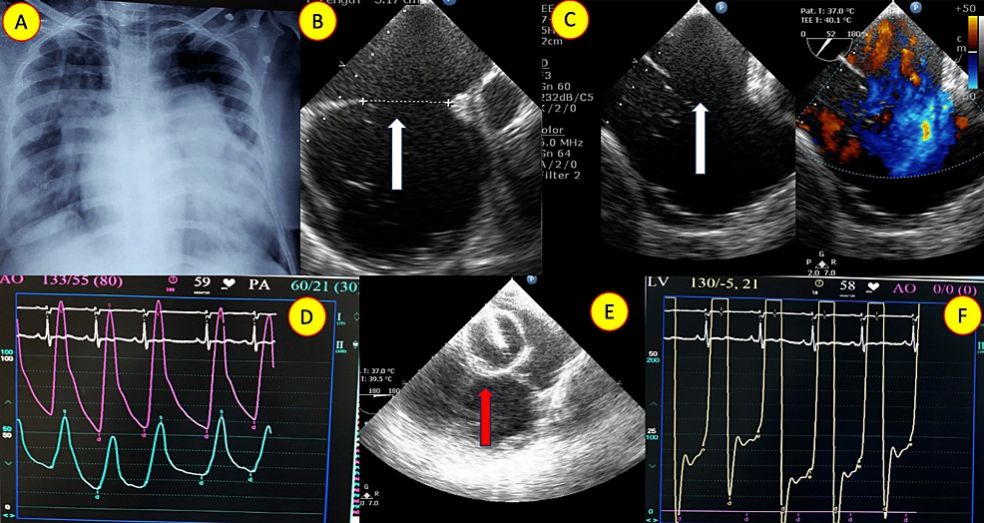
Secundum atrial septal defect with left ventricular diastolic dysfunction and pulmonary hypertension A: Chest X-ray (posterior-anterior view) showcasing significant cardiomegaly and a dilated main pulmonary artery. B, C: Transesophageal echocardiogram with color Doppler revealing a large secundum atrial septal defect (ASD) with bidirectional shunt (white arrow). D: Hemodynamic tracing demonstrating elevated pulmonary artery pressure. E: Balloon occlusion of ASD (red arrow) resulting in a substantial increase in left ventricular end-diastolic pressure (F).

A 46-year-old woman from the suburban region presented with severe symptoms of effort intolerance. She was found to have a large ASD with bidirectional shunting, left ventricular (LV) diastolic dysfunction, and moderate PH. During balloon occlusion of the ASD, the left ventricular end-diastolic pressure (LVEDP) increased markedly, indicating that immediate closure of the ASD was not advisable. Consequently, the patient was placed under medical observation and regular follow-up (Figure 5). This case underscores the crucial role of thorough hemodynamic evaluation during interventions in patients with PH. 

Case 6: idiopathic pulmonary artery dilation: unraveling the diagnostic puzzle

**Figure 6:**
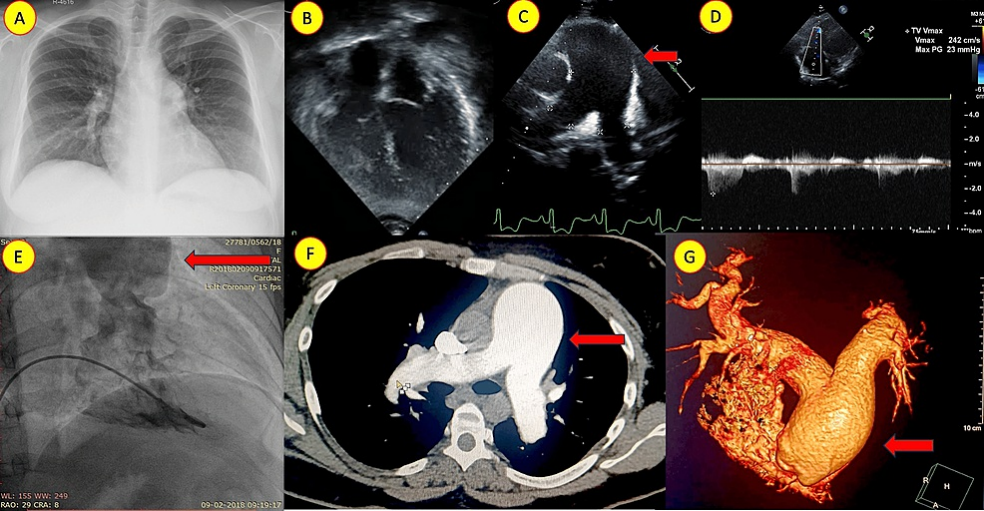
Idiopathic dilatation of the pulmonary artery A: Chest X-ray (posterior-anterior view) showing a cardiothoracic ratio of approximately 0.5 and a dilated main pulmonary artery. B: Echocardiogram in four-chamber view illustrating dominant left heart chambers. C: Parasternal short-axis view depicting dilated main and branch pulmonary arteries (red arrow). D: Continuous-wave Doppler of the tricuspid regurgitation jet showing mild elevation of right ventricular systolic pressure. E: Aneurysmally dilated pulmonary artery in the right ventricular angiogram and in computed tomography pulmonary angiography, two-dimensional. F: and three-dimensional reconstruction. G: is marked by red arrow.

A 41-year-old woman from Orissa State presented with a diagnosis of pulmonary hypertension. She had been receiving dual pulmonary vasodilator therapy for over five years without significant symptoms. However, an extensive evaluation at our institution revealed significantly dilated pulmonary arteries and elevated right ventricular systolic pressure by septal position. Upon comprehensive assessment, detailed cardiac catheterization confirmed the diagnosis of idiopathic pulmonary artery dilation. The pulmonary artery mean pressure was only 28 mm hg. Under close supervision, the patient's pulmonary vasodilators were gradually tapered off. A repeat cardiac catheterization three months later showed normalized pulmonary artery pressure. This case highlights the significance of accurate PH diagnosis and the challenges posed by improper PH labeling (Figure 6).

Three patients with an atrial septal defect (ASD) and a left-to-right shunt underwent successful device closure. However, they experienced moderate pulmonary hypertension (PH) and mild effort intolerance during the first four-week follow-up. Medications, including diuretics and Sildenafil, were administered for six to twelve weeks, after which pulmonary artery pressure normalized and symptoms resolved. Left ventricular (LV) diastolic dysfunction post-device closure was postulated as a contributing factor to PH, which gradually subsided (Table 5). This case highlights the need for continued detailed evaluation and surveillance of patients undergoing cardiac intervention with PH manifestations.

**Table 5:** Transient pulmonary hypertension in patients after device closure of an atrial septal defect

Number of patients	3
Average pulmonary artery systolic pressure during catheterization, (mm hg)	38
Average pulmonary artery mean pressure during catheterization (mm hg)	24
The average pulmonary blood flow to systemic blood flow ratio	2.3
The average pulmonary vascular resistance index (PVRI) during catheterization (wU.m2)	2.4
Estimated pulmonary artery systolic pressure after device closure of a trial septal defect in the follow up (mm hg)	61

Eight deaths were reported within the cohort, accounting for 8.6% of the patients (Table 6). Two deaths involved pregnancies complicated by congenital heart disease and severe PH. The loss of two pregnant women with severe PH reinforces the contraindication for pregnancy in patients with severe PH. The third case involved a young child with Down syndrome who had a history of elevated pulmonary pressure in infancy without structural heart disease. The child was initially treated with Sildenafil and was being weaned off the medication due to normalized pulmonary artery pressure. However, at two and a half years of age, the child developed acute respiratory symptoms and a moderate increase in pulmonary artery pressure attributed to lung issues. Unfortunately, delayed admission to the hospital resulted in the child's passing. This case underscores the importance of heightened awareness and comprehensive evaluation in managing pulmonary hypertension in individuals with Down syndrome. 

**Table 6:** Cause of death in different groups of pulmonary hypertension patients n: total number of patients, n1: number of patients in the prospective cohort, n2: number of patients in the retrospective cohort, %: % of the patients in the respective group.

Cause of death	Total cohort, n (%)	Group 1 (Prospective), n1 (%)	Group 2 (Retrospective), n2 (%)
Congenital heart disease	3 (3.22 %)	1 (1.69 %)	1 (2.94 %)
Pregnancy with congenital heart disease and pulmonary hypertension	2 (2.15 %)	0 (0%)	2 (5.88 %)
Idiopathic pulmonary hypertension (in a patient with Down Syndrome)	1 (1.07 %)	1 (1.69 %)	0 (0%)
Pulmonary hypertension with hepatic dysfunction and lung disease	1 (1.07 %)	0 (0%)	1 (2.94 %)
Pulmonary veno-occlusive disease	1 (1.07 %)	0 (0%)	1 (2.94 %)
Total number of deaths	8 (8.60 %)	2 (3.38 %)	6 (17.64 %)

Pulmonary hypertension treatment showed a good clinical response in 58.06% of patients within the cohort, with 32 in the prospective and 22 in the retrospective cohort. Seven patients, all from the prospective cohort, maintained a stable clinical status but experienced occasional destabilization episodes due mainly to superimposed lung infections requiring hospitalization. Unfortunately, 24 patients (25.80%) could not be reached by phone; 15 belonged to the retrospective cohort, primarily due to phone number changes, invalid contacts, or poor network reception. However, patients who responded to phone calls and recent follow-ups exhibited significant improvement in their functional class.

Within the cohort, three young ladies got married after initiating therapy. These cases included a patient with a ventricular septal defect (VSD) and severe PH who underwent successful surgery, another with systemic lupus erythematosus (SLE), and a third with patent ductus arteriosus (PDA) and severe PH. All three patients are doing well, highlighting the positive socio-personal implications of robust pulmonary hypertension care.

## Discussion

Pulmonary hypertension (PH) is characterized by elevated pulmonary vascular resistance, leading to gradual right ventricular failure, impaired quality of life, and reduced longevity. The PH spectrum encompasses a range of conditions, from the less prevalent but rapidly progressing pulmonary vasculopathy termed pulmonary arterial hypertension (PAH) to more common presentations linked to other causes like congenital heart disease (CHD).

The latest PH classification recognizes five distinct subtypes, each categorized based on underlying pathophysiological mechanisms [7,8]. Within the PULMOEAST study, a substantial 73.11% of patients were diagnosed with CHD, aligning with reported PH occurrences linked to CHD ranging between 11.3% and 16% in different studies [9]. The presence of untreated congenital heart conditions potentially contributes to the increased prevalence of PH in India, contrasting the pattern in developed nations where PH is primarily associated with left-heart diseases and less common conditions like idiopathic pulmonary arterial hypertension [1]. The higher proportion of CHD patients within our PH cohort was due to selective referrals to our congenital heart disease unit.

The PULMOEAST study was inspired by the PROKERALA study, a hospital-based registry focusing on investigating pulmonary hypertension's origins, treatment approaches, and outcomes. PROKERALA study utilized echocardiogram-derived pulmonary artery pressure to define pulmonary hypertension [10]. Building on Mukerjee et al.'s findings, where an echocardiographic tricuspid gradient surpassing 45 mmHg correlated with catheterization-diagnosed pulmonary hypertension in 97% of cases, PROKERALA adopted a more stringent threshold for accuracy [4,10]. Echocardiography-based registries, as seen in the developing world and exemplified by the PAPUCO registry in Sub-Saharan Africa, serve a crucial role in such settings [11].

In the indexed study, the majority of patients were categorized into functional classes 2 and 3. Conversely, other investigations like the KORPAH study reported a higher number of patients in classes 3 and 4, possibly indicating variations in distribution, clinical detection, and referral patterns across different geographical locations [12].

The PULMOEAST study employed a multi-level evaluation process for pulmonary hypertension, akin to the approach outlined in the study by Echazarreta et al. [7]. Initially, assessments included electrocardiograms (ECGs), chest X-rays, echocardiography, and general laboratory tests conducted in the majority of patients. These foundational tests are highly sensitive and widely available, though less specific. In the subsequent stage, the second-tier evaluation encompassed computed tomography and pulmonary angiography, performed in 32.25% of patients. The second-tier evaluations were primarily conducted in cases where the diagnosis was unclear or when there were suspicions of lung disease or pulmonary thromboembolism. This contrasts with the higher rates of CTPA reported by Echazarreta et al., ranging between 60% and 70% [7]. Additionally, the inclusion of the six-minute walk test (6MWT) was limited in the present study due to its recent introduction.

The third level of assessment involved cardiac catheterization. Diagnostic cardiac catheterization is considered the gold standard for confirming PH diagnosis, with low morbidity (1.1%) and mortality rates (0.055%) when performed by experienced practitioners [13]. Only 20 patients (21.5%) underwent cardiac catheterization in the indexed study, a lower proportion compared to other published studies. This reluctance might stem from patients' hesitance to undergo invasive tests due to associated risks, despite their significance in confirming diagnosis or aiding treatment response. Resource constraints in the region and the preference for simpler, non-invasive tests also contributed to the avoidance of invasive procedures.

Blood tests in the PULMOEAST study were conducted in two stages. Most patients underwent basic blood tests like a complete blood count, renal, liver, and thyroid function tests, and coagulation profiles. However, a smaller subset of patients, where the cause of PH was unclear, underwent extensive blood testing, including rheumatology profiles, serology for possible infection screening, etc. This extensive evaluation led to the detection of two cases of systemic lupus erythematosus (SLE), initially referred to as idiopathic PH. Blood tests in PH serve dual purposes: they aid in detecting or confirming causes in patients with secondary PH and can indicate potential organ-related issues. Comprehensive biochemical, hematological, and thyroid function tests are crucial for all patients. Liver function abnormalities could signal congestion, primary liver conditions, or treatment side effects. Elevated levels of NT-proBNP are associated with right ventricular dysfunction, predicting unfavorable outcomes. Tailored blood tests were performed in the majority of patients according to individual requirements, a trend also reported by Frost et al. [14].

The assessment of pulmonary function is crucial in evaluating PH patients. They can have underlying lung issues or lung disease as the cause of PH. Notably, a mild restrictive component is commonly observed in most patients with pulmonary hypertension [14]. Some patients in the study were referred by the pulmonary team for a PH assessment, as there were discrepancies in the clinical findings and echocardiographic evaluation done elsewhere. In the patients who were already diagnosed with PH in our unit, the diagnosis of respiratory involvement relied on clinical evaluation, an inadequate response to PH therapy, and findings from CTPA. Referring patients to respiratory consultants facilitated detailed pulmonary evaluations and care, leading to symptomatic improvements and better outcomes. The comprehensive cardiorespiratory evaluation helped optimize medications and outcomes for the patients.

Conducting multiple diagnostic tests during the initial evaluation and subsequent follow-ups posed challenges in the PULMOEAST study. Financial constraints among patients emerged as a significant barrier, restricting access to certain tests. The expense of NT proBNP, as highlighted by Echazarreta et al. [7], was a limiting factor in its use. Additionally, the higher risk associated with invasive tests contributed to their limited utilization.

Treatment for patients in the indexed study was personalized based on their diagnosis and clinical status. The foundational therapy primarily included loop diuretics and mineralocorticoid receptor antagonists for diuresis. A similar trend was observed in the African PH study [15], where loop diuretics were the most frequently prescribed medication (89% of all cases). In the PULMOEAST study, 11.82% of patients received anticoagulation primarily due to associated atrial arrhythmia and ventricular dysfunction. As for aspirin, it was administered to 22.58% of patients, while 3.2% received combination therapy. Three patients who underwent atrial septal defect device closure also received aspirin. The inclusion of aspirin is aimed at benefiting pulmonary hypertension patients [7]. The reasoning behind anticoagulation in idiopathic pulmonary hypertension cases lies in the high prevalence of in situ vascular thrombotic lesions, alongside coexisting coagulation, and fibrinolytic abnormalities. Additionally, there is a risk of venous thromboembolism attributed to heart failure and immobility. Echazarreta et al. reported anticoagulant use in 37% of patients with PH and in 69% of those in Group 4, showcasing a stark contrast to the usage pattern (81.6%) reported by an Argentine reference center registry for PH patients. This discrepancy highlights ongoing debates regarding the utilization of oral anticoagulation therapy [7,9]. In cases of ventricular dysfunction, a low-dose beta blocker was prescribed. Only a small subset of patients received digoxin for conditions like atrial arrhythmia and ventricular dysfunction, like findings reported by Echazarreta et al. [7].

The PULMOEAST study employed a diverse range of pulmonary vasodilator therapies (81.78%), encompassing monotherapy (27.95%) and dual therapy (53.76%), similar to findings in the RECOPILAR study [7]. Patients initially received a combination of Sildenafil and Bosentan, followed by a transition to a combination of Tadalafil and Ambrisentan to improve patient compliance, reduce rigorous hepatic function monitoring, and provide a simplified single-day dosing regimen. The Tadalafil-Ambrisentan combination demonstrated improved symptomatic relief among patients. Current guidelines emphasize aggressive treatment strategies for pulmonary hypertension, advocating for the use of combination therapies. Previous reports indicate combination therapy rates ranging from 17% to 44% at different stages post-diagnosis [9]. Three patients in our cohort underwent intervention for shunt lesions, two with successful surgical closures and one with a fenestrated closure of an atrial septal defect using a custom-made septal occluder. A detailed hemodynamic assessment guided the decision-making process. Fenestrated closure of shunt lesions is a viable option for selected patients with pulmonary hypertension and a significant left-to-right shunt [16].

In the PULMOEAST study, most patients were referred with moderate or severe pulmonary hypertension without thorough evaluation to determine the potential cause and categorization of functional class. Many patients were unaware of the diagnosis and its implications. This underutilization of PH-targeted care in eastern India stems from various factors, including limited healthcare access, delayed healthcare seeking, the subtle nature of PH symptoms causing delayed detection, a lack of awareness among primary care physicians, constrained access to echocardiography services and specialized care, drug availability, side effects, and cost. Similar challenges have been reported by researchers in other low- and middle-income countries [9,12,15,17].

Detailed counseling of patients and their families regarding the diagnosis, nature of the disease, management plan, and importance of compliance improved outcomes in our study, particularly within the prospective cohort compared to the retrospective cohort. Emphasizing initial symptom relief through optimal medical care, followed by basic PH investigations and secondary cause assessment, proved to be an effective strategy. Collaboration between the pulmonary and cardiovascular teams facilitated more coordinated cardio-pulmonary therapy, enhancing patient compliance and perceived benefits. Effective record maintenance within the prospective cohort enabled close monitoring of treatments, and dropout rates within this group remained insignificant. An effective approach to managing pulmonary PH involves a comprehensive evaluation and treatment conducted by a multidisciplinary team. Persistent efforts to raise awareness among both the general public and healthcare professionals will greatly aid in our endeavor to treat pulmonary hypertension [9,18].

Limitations of the study 

The study confronted several limitations that warrant acknowledgment. The primary referral pattern to our institution resulted in an overrepresentation of Group 1 PH patients linked to congenital heart disease, potentially skewing our findings and not thoroughly reflecting the entire PH spectrum within our hospital. Additionally, the retrospective cohort, dependent on available records, might not encompass all diagnosed PH cases, introducing potential selection bias. The management decisions, particularly regarding cardiac components, were predominantly influenced by the lead congenital heart disease specialist, potentially introducing a degree of bias in diagnosis and management strategies. Moreover, the transition of our hospital's patient data management software twice during the study duration posed challenges in retrieving comprehensive data, which could have impacted the inclusivity of the study sample.

A subset of our cohort did not undergo diagnostic cardiac catheterization, considered the gold standard for PH diagnosis, which contrasts with established studies and could have affected the robustness of our diagnostic approach. The delayed commencement of the six-minute walk test, a recognized method for monitoring functional class, limited our ability to comprehensively assess functional status. Future analyses integrating this test could offer valuable insights.

Furthermore, the limitations encountered in this study reflect the complexities inherent in conducting research in resource-constrained settings. Relying on echocardiography rather than right heart catheterization for diagnosis, while practical in such settings, may have constrained the depth of our analysis, particularly within specific subsets of PH cases. The constrained number of cases further hindered our ability to conduct a more thorough examination within these subsets.

## Conclusions

Pulmonary hypertension poses a significant health concern in eastern India, as revealed by the PULMOEAST study. This research identified congenital heart disease as the primary culprit for PH in this population. Echocardiography, a readily available and effective tool, proved crucial for initial and follow-up evaluations, especially in resource-scarce settings. Encouragingly, most patients exhibited notable clinical improvement with supportive medications and pulmonary vasodilators. However, diverse PH etiologies, limited access to PH-specific resources, and a lack of widespread awareness remain substantial challenges.

The PULMOEAST study's findings underscore the need for refined diagnostic approaches. Exploring alternatives to gold-standard invasive procedures like cardiac catheterization could improve the accessibility and affordability of PH care. Cost-effective management strategies like affordable medications and accessible treatment options are crucial to ensuring wider patient reach. Collaborative care initiatives between cardiologists, pulmonologists, and other specialists can provide comprehensive care and improve PH patient outcomes. Enhanced patient education by raising awareness about PH, diagnosis, and management options within the community empowers individuals to seek timely care. By addressing these challenges and implementing the proposed strategies, PH care in eastern India can be significantly optimized, leading to improved health outcomes for patients in the region. The PULMOEAST study offers valuable insights into the PH landscape of eastern India and lays the groundwork for future research and improved PH care in the region.
